# Designing Nanoconfined LiBH_4_ for Solid-State Electrolytes

**DOI:** 10.3389/fchem.2022.866959

**Published:** 2022-04-08

**Authors:** Suwarno Suwarno, Angeloclaudio Nale, Putu Suwarta, Ika Dewi Wijayanti, Mohammad Ismail

**Affiliations:** ^1^ Department of Mechanical Engineering, Institut Teknologi Sepuluh Nopember (ITS), Surabaya, Indonesia; ^2^ “Section of Chemistry for Technologies”, Department of Industrial Engineering, University of Padova, Padova, Italy; ^3^ Energy Storage Research Group, Faculty of Ocean Engineering Technology and Informatics, Universiti Malaysia Terengganu, Kuala Terengganu, Malaysia

**Keywords:** hydrides, lithium borohydrides, battery, electrolyte, solid-state, batteries and energy storage

## Abstract

Solid-state electrolytes are necessary for high-density and safe lithium-ion batteries. Lithium borohydride (LiBH_4_) is one of the hydride compounds that shows promising candidates for solid-state electrolytes and enables all-solid-state batteries. LiBH_4_ has good wetting properties and preferable mechanical properties when used in battery cells. The Li-ion conduction in LiBH_4_ can be modified with nanoconfinement as a result of distinct properties on the interfaces. The ion conductivities can be modified further by choosing property support materials, i.e., composition, textural properties, and surface chemistry. The present work briefly reviews the Li-ion conduction in nanoconfined LiBH_4_. A future perspective on the development of LiBH_4_ as a solid-state electrolyte is further elaborated in the last section.

## Introduction

The availability and widespread use of clean and sustainable energy are a global challenge in the present century. Clean energy can be obtained from converting renewable energy sources such as solar, waves, and wind. However, most renewable energy sources are intermittent, and thus, storage technology is required to maximize widespread usage. Energy storage uses various technologies from mechanical, chemical, and electrochemical storage. Batteries and fuel cells are the two electrochemical storage technologies currently employed for mobile and stationary energy storage and have shown the potential to be developed further. For batteries, specific energy density, in terms of both weight and volume, is an essential parameter besides the costs and abundance of the element. Currently, the battery used in electric cars uses lithium-ion technology, which has a capacity ranging from 100 to 300 Wh/kg based on the intercalation electrode concepts (Schmuch et al., 2018). Increasing the energy density up to 10 times the current technology is expected based on the initial investigation and theoretical calculation using Li as an anode instead of carbon or silicon materials (Adelhelm et al., 2015). The advanced lithium cell battery has an energy density of around 260 Wh/kg using state-of-the-art intercalation electrodes, for example, in the NCA/Si–C cell (Schmuch et al., 2018). Nevertheless, there is enormous room for improvement in increasing the energy density, such as using the lithium anode and sulfur or lithium–air chemistry (Bruce et al., 2012).

Advanced development is enabled by a solid-state electrolyte that has the potential to overcome the well-known battery challenge, i.e., the formation of lithium dendrite. In addition to this safety issue, a high-energy density battery can be realized. Enabling a high-density battery and stable cell operation requires high ionic conductivity, a large potential window, and excellent wetting properties to achieve intimate contact with the electrode. To date, several classes of inorganic materials can be used for ionic conducting materials, such as oxide base LISICON-like, NASICON-like, perovskite, and hydride compound materials, with their advantages and disadvantages when implemented in the battery system.

A considerable effort to increase ionic conduction has been explored; this includes designing an alloying element that provides a tunable lattice volume, energy landscape, and phonon vibration (Krauskopf et al., 2018; Muy et al., 2018). Another approach is materials confinement to benefit from the support materials’ interfacial properties. The journey for finding a new class of electrolytes is ongoing; recently, a boron hydride class has been found to have high ionic conductivity and stability to be used in the Li–S system (Kim et al., 2019). In addition to the properties within the crystal, the interface properties such as grain boundaries play a role in the bulk ionic mobility due to space charge developing at the interface (Yu and Siegel, 2017).

## Nanoconfined Hydride

LiBH_4_ is thermodynamically stable with an enthalpy of formation of 78 kJ/mol H_2_. The compound desorbs up to 18.5 wt% H and thus is well known as a promising material for hydrogen storage (Schlapbach and Züttel, 2001). However, hydrogen can only be released at above 673 K *via* various desorption steps hindering further practical application. LiBH_4_ experiences polymorph transformation from the orthorhombic phase into the hexagonal type structure. At room temperature, the crystalline phase LiBH_4_ has an orthorhombic structure. It transforms to the hexagonal phase at 383 K. Interestingly, for the hexagonal phase, Li^+^ in LiBH_4_ is highly mobile, resulting in high ionic conductivity, up to 10^−3^ S/cm, which is several orders of magnitude compared to that of the low-temperature phase (Matsuo et al., 2007) and has approximately 6 V stability. Further study using temperature- and frequency-dependent nuclear magnetic resonance (NMR) spectroscopy showed low dimensionality of Li^+^ in LiBH_4_ (Epp and Wilkening, 2010). Therefore, this material is promising for the solid-state ionic conductor in Li-ion batteries. These lead to further research on the application of LiBH_4_ as superionic conductors. Several light hydride materials based on boron have ionic conductivity (Callini et al., 2016; Mohtadi and Orimo, 2017; Guzik et al., 2019; Mohtadi, 2020). Lithium borohydride (LiBH_4_) consists of Li^+^ cation and [BH_4_]^-^ anion complexes.

Various routes/methods have been explored in search of improvement of the properties of LiBH_4_, and several research groups have been reviewed on this topic (Ley et al., 2014; Bannenberg et al., 2020). Since it has low surface tension, LiBH_4_ infiltrates porous support such as SBA 15, in which the key was adding hydrogen pressure during the process (Ngene et al., 2010). The wetting properties lead to an excellent capillary infiltration on any porous material such as carbon materials and porous oxides (de Jongh and Eggenhuisen, 2013). This led to nanoconfined materials for hydrogen storage with enhanced properties (de Jongh and Adelhelm, 2010). Another strategy is to confine the metal hydrides in porous matrices, which can be achieved *via* melt infiltration (Ngene et al., 2010; de Jongh and Eggenhuisen, 2013).

Recently, fast ionic conductivity in silica-confined LiBH_4_ has been identified (Blanchard et al., 2014). The reason for that is an increased mobility of the [BH_4_]^-^ units and possibly Li^+^ in the confined phase (Verkuijlen et al., 2012). Earlier neutron scattering experiments and NMR suggested that the confined materials comprise a highly mobile phase, which was argued to be a consequence of the confinement’s stress. It has also been recommended that the confined LiBH_4_ is composed of two distinct fractions of LiBH_4_: fast mobility in the interface and slow mobility at the core (Liu et al., 2013; Verdal et al., 2013; Breuer et al., 2018).

**FIGURE 1:**
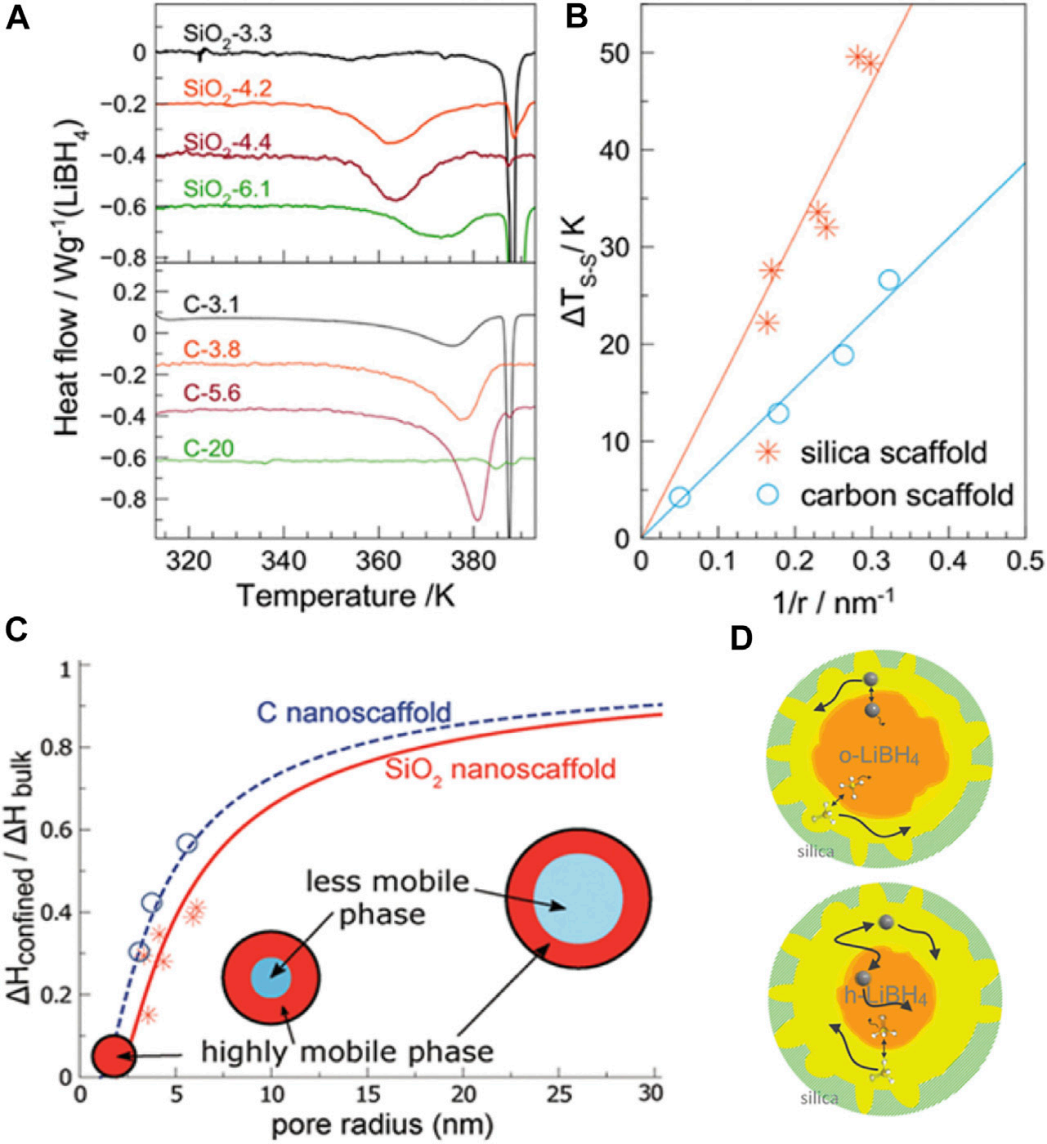
Effect of nanoconfinement on LiBH_4_: **(A)** differential scanning calorimetry profile showing the other bottom of the profile that originated from confined LiBH_4_ at a lower temperature than that of the bulk; **(B)** transformation temperature depression (ΔT) as a function of the pore size for two different support materials; **(C)** ratio of enthalpy from confined and bulk LiBH_4_ in the composite for fitting the layer thickness of the mobile phase; **(D)** schematic of mobile phase dynamics from analyzing the NMR data (Suwarno et al., 2017; Lambregts et al., 2019).

The Li^+^ conductivity is structurally dependent and reaches up to 1 × 10^−3^ S/cm, at high temperature and when LiBH_4_ is in its hexagonal phase. This value is close to that of liquid electrolytes primarily used in Li-ion batteries (Blanchard et al., 2015). The high conductivity has been explained because of the formation of highly mobile liquid-like LiBH_4_ close to the surface of the pores (Suwarno et al., 2017). Figures 1A,B show the pore size dependence of the transition temperature of the confined LiBH_4_. The interfacial thickness layer has been estimated to be 1.94 and 1.41 nm for silicon and carbon support materials (Figure 1C). Later studies using NMR confirmed the existence of two phases for the high mobility of lithium, as shown in Figure 1D (Lambregts et al., 2019).

## Discussion

The beneficial effect of LiBH_4_ as a potential solid-state electrolyte that can be realized is the low melting of LiBH_4_ and low surface tension in a liquid phase. Lower melting temperature and surface tension can be easier for fabrication and integration in ASSL batteries (Xiao et al., 2021). In addition, the softness increases the interfacial properties of the battery cell compared to the ceramic-based electrolyte. Another essential feature of the solid-state electrolyte used in the cell is the thermodynamic stability. A preliminary experiment showed that the voltage window was at 5–6 V, even though a recent investigation revealed that LiBH_4_ works well at 2–3 V in the Li–S system. The most important requirement of solid electrolytes is fast ionic conduction at room temperature (10^−3^ S/cm) to compete with the current liquid electrolyte.

**FIGURE 2:**
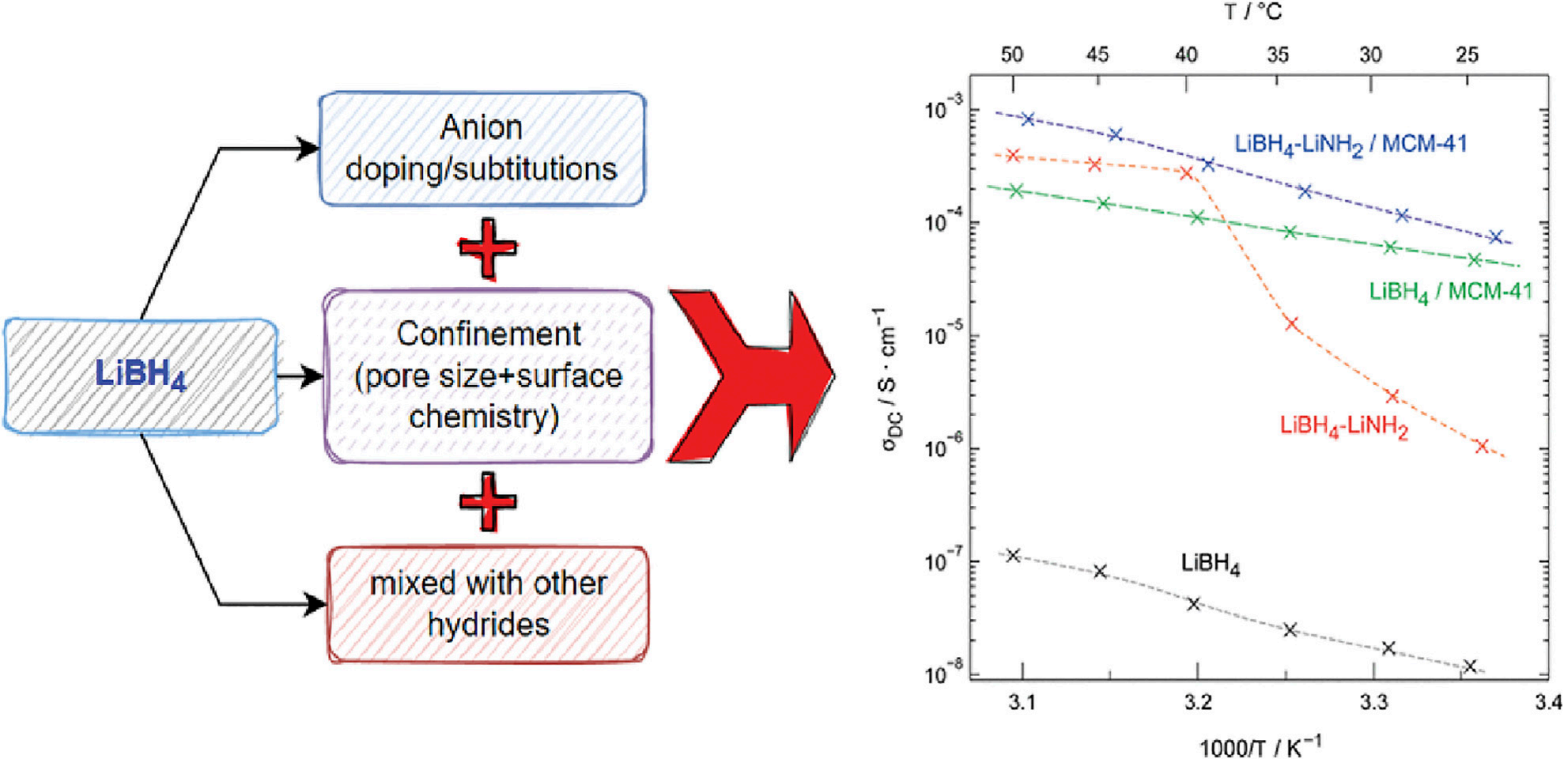
Summary of various strategies for designing enhanced Li^+^ conductivity in LiBH_4_ and the Li^+^ conductivity plot from the previously reported study. The conductivity plot is taken from the study of Zettl et al. (2020).

The Li^+^ mobility in LiBH_4_ can be modified by using various methods. Figure 2 (left side) summarizes the different methods to increase the Li conductivity, and the initial attempt was by using anion doping. This can be achieved, for example, by doping with LiCl. The most successful was the addition of LiI (Maekawa et al., 2013). This earlier experiment showed that doping the anion dynamics enhanced the Li-ion conduction (Maekawa et al., 2013; Sveinbjörnsson et al., 2013). The second approach is nanoconfinement using support materials such as nanoporous silica or alumina. Since the hexagonal high-temperature phase LiBH_4_ has high conductivity, it is rational to stabilize the high-temperature phase by confinement. This comes from the Gibbs–Thomson relation of the transformation temperature dependent on the particle size. The third approach is a composite of two Li-based hydrides such as LiNH_2_.

The initial experiment on the nanoconfined LiBH_4_ mobility used NMR, which showed that the highly mobile phase is suggested from within a thin layer of high-temperature phase that is stable because of the stress by the confinement in SiO_2_ porous materials (Verkuijlen et al., 2010; Blanchard et al., 2015). The role of an interface has also been shown in the LiBH_4_/Al_2_O composite (Epp and Wilkening, 2013). Thus, the thickness of the interfacial layer could play a significant role in the conductivity. The possibility to tune a well-defined geometry and pore radius of ordered porous silica allowed further research to access the thickness interfacial layer. Furthermore, the surface effect is indicated by comparing silica and carbon to elucidate the interface effect. This was an initial attempt to study the surface effect in addition to the pore size dependency on controlling properties of LiBH_4_. A later study showed a significant impact on the surface of silica, in which the silanol group played/had a prominent role in Li^+^ conduction (Ngene et al., 2019). Experiments using various oxides to study the effects of support on the Li-ion conduction show that MgO has a better role in increasing the Li^+^ conductivity (Gulino et al., 2020, 2021). Figure 2 shows several results from an approach or combination of the Li^+^ conductivities to highlight a summary of results. As shown in Figure 2 (right), combining two approaches has also been tried, which showed a promising result. Experiments on the nanoconfined LiBH_4_–LiI in SiO_2_ support further improvement of ionic conductivity and decreased activation energy for Li^+^ diffusion (Zettl et al., 2020). The Li^+^ conductivity enhancement was also observed in LiBH_4_–LiNH_2_ confined in mesoporous silica (MCM-41). Surprisingly, in the confined LiBH_4_–LiNH_2_ composites, the surface effect is less dominant than that in LiBH_4_ alone (Kort et al., 2020). The recent study of the composites containing LiBH_4_–LiI/Al_2_O_3_ and LiBH_4_/Al_2_O_3_ highlighted the crucial effect of the insulator–conductor interface in creating the path for fast ion conduction (Zettl et al., 2021). Thus, further exploration of combining various approaches seems promising to enhance the Li^+^ conductivity in LiBH_4_.

## Conclusion

Lithium borohydride has been widely studied in the last decade and is a promising candidate for solid-state electrolytes used in all-solid-state batteries. The hexagonal phase can be stabilized by nanoconfinement, and the interfacial properties between LiBH_4_ and the support materials determine the Li^+^ conductivity. Various approaches combining nanoconfinement with other approaches such as anion substitutions and surface modification yield an increase in ionic conductivities. However, the effect seems different between single-phase LiBH_4_ and when it is mixed with LiNH_2_. The effect of the pore volume is dominant rather than the surface effects in the confined LiBH_4_+LiNH_2_. Further studies on the impact of LiBH_4_ doping and textural properties of the support materials on the Li^+^ conductivity can be the direction of future explorations.
